# A novel scaling methodology to reduce the biases associated with missing data from commercial activity monitors

**DOI:** 10.1371/journal.pone.0235144

**Published:** 2020-06-24

**Authors:** R. O’Driscoll, J. Turicchi, C. Duarte, J. Michalowska, S. C. Larsen, A. L. Palmeira, B. L. Heitmann, G. W. Horgan, R. J. Stubbs

**Affiliations:** 1 Appetite Control and Energy Balance Group, School of Psychology, University of Leeds, Leeds, United Kingdom; 2 Department of Treatment of Obesity, Metabolic Disorders and Clinical Dietetics, Medical Faculty, Poznan University of Medical Sciences, Poznan, Poland; 3 Research Unit for Dietary Studies, The Parker Institute, Bispebjerg and Frederiksberg Hospital, The Capital Region, Denmark; 4 Faculdade de Motricidade Humana, Universidade de Lisboa, Lisbon, Portugal; 5 Universidade Lusófona, Lisbon, Portugal; 6 Department of Public Health, Section for General Medicine, Copenhagen University, Copenhagen, Denmark; 7 Charles Perkins Centre, The Boden Institute, University of Sydney, Sydney, Australia; 8 Biomathematics & Statistics Scotland, Aberdeen, United Kingdom; Baylor College of Medicine, UNITED STATES

## Abstract

**Background:**

Commercial physical activity monitors have wide utility in the assessment of physical activity in research and clinical settings, however, the removal of devices results in missing data and has the potential to bias study conclusions. This study aimed to evaluate methods to address missingness in data collected from commercial activity monitors.

**Methods:**

This study utilised 1526 days of near complete data from 109 adults participating in a European weight loss maintenance study (NoHoW). We conducted simulation experiments to test a novel scaling methodology (NoHoW method) and alternative imputation strategies (overall/individual mean imputation, overall/individual multiple imputation, Kalman imputation and random forest imputation). Methods were compared for hourly, daily and 14-day physical activity estimates for steps, total daily energy expenditure (TDEE) and time in physical activity categories. In a second simulation study, individual multiple imputation, Kalman imputation and the NoHoW method were tested at different positions and quantities of missingness. Equivalence testing and root mean squared error (RMSE) were used to evaluate the ability of each of the strategies relative to the true data.

**Results:**

The NoHoW method, Kalman imputation and multiple imputation methods remained statistically equivalent (p<0.05) for all physical activity metrics at the 14-day level. In the second simulation study, RMSE tended to increase with increased missingness. Multiple imputation showed the smallest RMSE for Steps and TDEE at lower levels of missingness (<19%) and the Kalman and NoHoW methods were generally superior for imputing time in physical activity categories.

**Conclusion:**

Individual centred imputation approaches (NoHoW method, Kalman imputation and individual Multiple imputation) offer an effective means to reduce the biases associated with missing data from activity monitors and maximise data retention.

## Introduction

Participation in physical activity and limiting sedentary behaviours is associated with increased total energy expenditure and potentially beneficial homeostatic matching of energy intake to energy expenditure [1]. As such, more active lifestyles are associated with a reduced risk of obesity [2], weight loss and prevention of weight regain following weight loss [3–5], as evidence suggests that weight maintenance is more readily achieved at higher degrees of energy flux [6]. Thus, the accurate and precise quantification of physical activity behaviours is critical to the study of overweight, obesity and associated comorbidities.

Accelerometery-based measures of physical activity have been available for a number of years [7]. Their objective nature offers a significant advantage over questionnaire-based assessments, which are biased by misreporting [8]. In current activity monitors, tri-axial piezoelectric sensors detect acceleration in anteroposterior, mediolateral and vertical axes and are used to objectively quantify human movement [9]. Technological advances in terms of size, data aggregation/storage capabilities and the associated fall in cost facilitates the use of tri-axial accelerometers in most new devices [9], as opposed to the uni-axial [10], bi-axial accelerometers [11] and burdensome battery packs required for earlier devices [12]. Taken together, these advances mean that it is increasingly feasible to objectively and continuously monitor the intra-day physical activity patterns of large groups of participants.

A well-recognised phenomenon in accelerometer research is missing data [13] attributable to behavioural (removal for aesthetic reasons) and non-behavioural reasons (device technical failures, charging). Non-wear time in accelerometers has previously been detected by defining periods in which the signal of acceleration in each axis falls below a threshold for some period of time, often a predefined period between 10–120 minutes [14,15]. Researchers then permit a maximum amount of non-wear time per day, which may be up to 14 hours [16]. The aim of defining such a period is to determine the amount of missing data which minimally influences the inferences of the study [17]. It is also common to define a minimum number of valid days within a measurement period and if these criteria are met, an average or total value for physical activity metrics can be estimated [18,19].

Missing accelerometer data may detrimentally influence the conclusions of a study in a number of ways. If physical activity summaries are calculated from incomplete data, true physical activity may be under-estimated (depending on the assumptions made about missing data). If missing periods occur in individuals that differ behaviourally or demographically from those with more complete data then the generalisability of the study’s conclusions may be compromised [20]. A range of strategies have been developed with the aim of limiting the bias introduced by missing accelerometer data [21]. These methods make use of the observed (non-missing) data to build predictive models of missing data points and have utilised mean imputation [22], combined multivariate strategies [23,24] or normalisation by the amount of wear-time [25,26].

Commercial activity monitors are increasingly prevalent in research environments and may be utilised in large cohorts and over long durations for assessment of physical activity. Commercial activity monitors are cloud-connected, facilitating the assessment of physical activity for longer time periods than research-grade equivalents (i.e. Actigraph GT3-x), which typically measure physical activity maximally over a single week [27]. Commercial activity monitors are also increasingly equipped with heart rate monitoring devices [28], which can facilitate the estimation of the relative intensity of physical activity or energy expenditure, through heart rate reserve (HRR) or flex methodologies [29–32] but also creates different patterns of missingness. For example, missing data may be identified through loss of contact with the wrist (and therefore no measured heart rate), inferring that the device has most likely been removed. This results in the detection of smaller windows of removal, compared to longer periods used when accelerometer signal is the determinant of missingness [14,15]. These differences highlight an important need to develop methods to limit the bias associated with missing data from these devices. There has been no attempt to develop or apply imputation methodologies to commercially available multisensory activity monitors (i.e. Fitbit charge 2; FC2).

The purpose of the present study is to propose and evaluate a methodology designed to minimise the bias introduced by missing data collected from a commercial activity monitor (FC2). Firstly, we conducted a series of intra-class correlation analyses to investigate the minimum data required to achieve a reasonably non-biased aggregation of physical activity data collected by a FC2. Next, the results of autocorrelation analyses are presented, which serve as the rationale for the development of a method which scales temporally proximate data to produce summaries over a given measurement period. Lastly, in a series of simulation experiments using real datasets with simulated missingness, we compared the performance of the proposed methodology to alternative imputation strategies.

## Materials and methods

### Participants

Data were collected as part of the NoHoW trial (ISRCTN88405328), an 18-month randomised 2x2 controlled trial testing the efficacy of an ICT based toolkit for weight loss maintenance across three European centres: United Kingdom, (Leeds), Denmark (Copenhagen), and Portugal (Lisbon). The NoHoW study received funding from the European Union’s Horizon 2020 research and innovation programme (grant agreement number: 643309). The study was conducted in accordance with the Helsinki Declaration and ethical approval has been granted by local institutional ethics committees at the Universities of Leeds (17–0082; 27-Feb-2017), Lisbon (17/2016; 20-Feb-2017) and the Capital Region of Denmark (H-16030495; 8-Mar-2017) and all participants provided informed consent to have their data used for research purposes by this research team. Full details of the trial protocol have been published previously [33]. The NoHoW trial recruited 1,627 participants and some of the observational work reported in this study utilised the entire sample of NoHoW participants and when this is the case, this is specified in the manuscript.

For the simulation experiments conducted in this study, FC2 data from 109 participants each wearing a FC2 for 14 days (minutes = 2,197,440, hours = 36,624, days = 1526) were used. This sample was selected based on the quantity of non-wear time (<2.5% data missing within the first 14 days). Utilising a sample with minimal degrees of missingness allows ‘true’, near-complete data to be held back for comparison with imputation methods.

### Fitbit Charge 2 (FC2)

All participants enrolled in the NoHoW trial were provided with a FC2 (FC2; Fitbit Inc, San Francisco, CA, USA). The FC2 is a wrist-worn activity monitor which derives estimates of energy expenditure and physical activity based on data obtained from incorporated sensors and proprietary algorithms. The FC2 estimates of heart rate are obtained through a patented technology called ‘PurePulse’, which uses light-emitting diodes to monitor blood volume [28]. Data are aggregated to the minute-level and synced via the Fitbit mobile application to Fitbit servers through an application programming interface. In the present study, non-wear time is defined by the absence of a heart rate measure and all devices were set to ‘auto’ mode by default, which ensured that no heart rate reading was transmitted when the device was not on the wrist.

### Autocorrelation analyses

**Fig 1:**
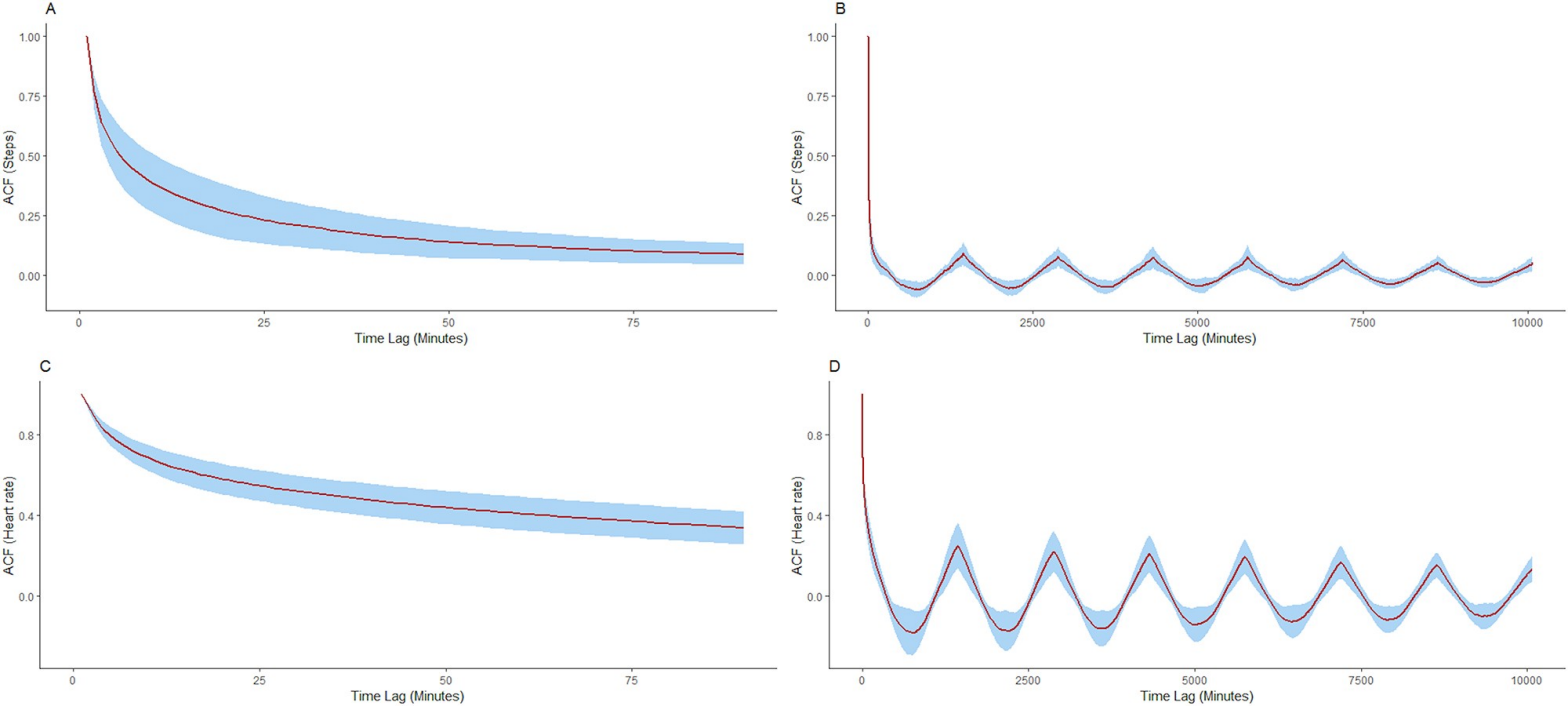
Autocorrelation (ACF) values for steps with time lags of 90 minutes (A), 10,080 minutes (B) and heart rate with time lags of 90 minutes (C) and 10,080 minutes (D). Average ACF values are shown in red and the blue ribbon represents ± 1 standard deviation.

The algorithm proposed in this study was initially based on a series of autocorrelation analyses which are presented below. In autocorrelation analyses, the correlation between values in the time series are computed as a function of the time lag between them, defined in minutes in this case. For these analyses we calculated the autocorrelation value for all time lags of up to 7 days (10080 minutes) for each participant individually, thus indicating time points within a week with the highest correlation. Fig 1 illustrates the autocorrelation for steps and heart rate for 90 minutes and 10081 minutes, respectively.

The average of the autocorrelation values (ACF) reached within 60 minutes for steps are: 15 mins: ACF = 0.31, 30 mins: ACF = 0.21, 45 mins: ACF = 0.15, 60 mins: ACF = 0.12, comparatively, heart rate values are higher: 15 mins: ACF = 0.62, 30 mins: ACF = 0.52, 45 mins: ACF = 0.46, 60 mins: ACF = 0.41. Although there is evidence of periodic patterns on subsequent days, the value does not exceed ACF = 0.09 for steps, which is observed at a lag of 1441 minutes and ACF = 0.25 is observed for heart rate at 1440 minutes, the differences in these values are likely attributable to the stochastic nature of steps when compared to heart rate. Notably, the value at 10081 mins (7 days) is ACF = 0.05 for steps and ACF = 0.13 for heart rate. Thus, the greatest autocorrelation values are observed locally for both steps and heart rate.

### Wear time requirements

**Fig 2:**
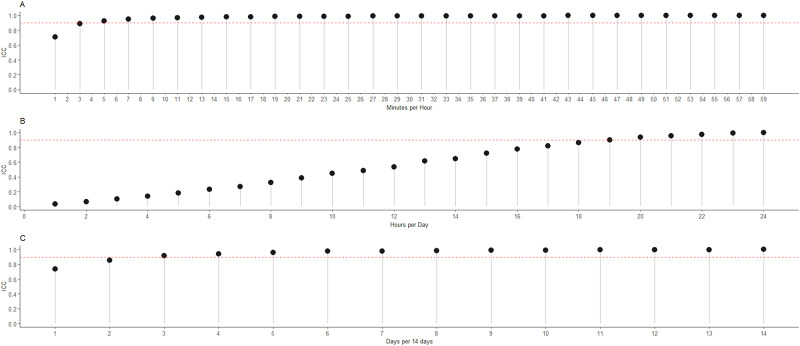
Intraclass correlations (ICC) for incrementally deleted data and ‘true’ data. Data are presented for scaled minutes per hour (A), for hours per day (B) and for number of days per 14 days (C).

In order to investigate the minimum amount of wear-time required for a valid hour, day or 14-day period, intraclass correlation (ICC) analyses were conducted, as ICC is a widely used and accepted means of determining measurement agreement [34]. In each of these experiments, data were deleted incrementally and at random and the ICC was calculated between the partially deleted data and the ‘true’ steps at each increment. An ICC threshold of 0.9 was used as the selection criterion to represent 10% similarity of true values [18]. We first investigated the minimum time required within a single hour with adjustment for wear time, and thus the remaining data was divided by the proportion of the wear time and this adjusted value was used for ICC analyses. In the daily and 14-day analyses, adjustments for wear time were not made. For all analyses, two-way mixed-effects agreement models were used [34] and this was conducted with the ‘icc’ function from the ‘rel’ package in R. Fig 2a demonstrates that if 5 minutes of data are present and scaled to 60 minutes, the ICC threshold of 0.9 is reached. In the daily analysis, the ICC threshold was met at 18–19 hours per day (Fig 2b). It is important to note that our ICC comparisons for each day include non-scaled data despite using scaled data in our algorithm (outlined below). When scaling by the proportion of wear time per day, the number of hours required will be lower. We utilise 18 hours to ensure that true data are available from different parts of the day (i.e. morning, afternoon, evening) and this is a conservative requirement in line with previous research [35]. To establish minimum 14-day requirements, the ICC threshold was met at 3 days (Fig 2c). For the final algorithm, we required 4 days including at least one weekend day as the minimum criteria for inclusion, owing to the potential for differential patterns of physical activity between weekdays and weekend days [36].

### NoHoW algorithm

Based on these analyses we propose a scaling algorithm, referred to from hereon as the ‘NoHoW algorithm’ as follows:

1. *If* non-missing minutes per hour < 5 *then* remove hour from dataset *else* sum available minutes to provide hourly total
2. Divide the number of available minutes per hour by 60 to give the proportion of wear time per hour
3. Divide hourly total by the proportion of wear time per hour to provide a scaled hourly total
4. *If* available hours per day < 18 *then* remove day from dataset *else* sum all available hours to give daily total
5. Divide the number of available hours by 24 to give proportion of wear time per day
6. Divide daily total by the proportion of wear time per day to provide a scaled daily total
7. *If* available days per 14 days < 4 *or* < 1 weekend day *then* remove 14-day period from dataset *else* average all valid days

### Simulation experiments

In order to test the algorithm, we performed two simulation experiments. In the first experiment, we tested traditional imputation methods as well as the proposed algorithm. This was achieved by creating datasets with simulated missingness from each of the included participant’s true data and holding back this true data to be compared to the imputed datasets. The time point at which the data were removed was random and the length of each deleted period was uniformly sampled between one and 120 minutes in duration. The decision to insert missing data at random positions was informed by observing the proportion of missing FC2 data for each hour in the first 14 days of the NoHoW study, on average 22.83% was missing with a range of 21.1% at 13:00–13:59 to 25.96% at 23:00–23:59 (S1 Fig). To determine the length of missing periods in this study, we quantified the length of each missing period in in the first 14 days of the NoHoW study, where the length was less than an entire day (1440 minutes). Of the 146,165 missing periods, 139,213 (95.24%) were less than 60 minutes and 3882 (2.7%) were greater than 120 minutes (S2 Fig), thus we set 120 minutes as the upper limit for the length of insertions. The final parameter in the missing data algorithm was the number of missing periods, which was set to 40. This resulted in the amount of missing data per day being 13.7% (11.76% inserted) on average and ranging up to 44.4% (36.81% inserted) in simulation study 1.

Utilising the same simulated missing datasets, our first simulation study tested the methodologies below for dealing with missing data.

### Removal

The effect of no imputation or adjustment strategy was demonstrated by simply reporting the physical activity summaries for the simulated missing datasets.

#### Mean imputation

Missing data were imputed with the i) mean of all the remaining data and ii) with the mean of the individuals remaining data. This was conducted with the *Hmisc* package in R.

#### Random forest imputation

We performed random forest imputation, utilising the ‘missForest’ package in R. This is a non-parametric imputation method, which implements the original random forest algorithm [37]. We performed random forest imputation to predict the missing values for steps, heart rate and calories on each participants data using weekday and hour as observed, non-missing variables. Hyperparameters were selected with consideration of computational feasibility; We utilised 100 trees in each forest, the number of randomly sampled variables at each split was set to the square root of the number of variables and the maximum number of iterations was set to 5.

### Multiple imputation

We tested multiple imputation with the use of bootstrapping and predictive mean matching utilising i) the entire sample and ii) individual-level data. In the case of the overall model, we utilised age, gender and day of the week as covariates, as they have previously been shown to be associated with differential patterns of physical activity [18,38]. In the individual models, hour of the day was used as an additional covariate. An advantage of multiple imputation is the repetition of the imputation process thus attempting to address the uncertainty associated with a single imputation. We utilised 5 imputations in the overall model, and in the individual level model we utilised 7 imputations. Multiple imputation was implemented with the *Hmisc* package in R.

### Kalman imputation

Lastly, we tested Kalman smoothing imputation using a structural time series model. Kalman imputation was implemented with the *imputeTS* package in R to impute caloric expenditure, steps and heart rate.

### Simulation study 2

In simulation study 2, we investigated how the bias introduced by the NoHoW algorithm, Kalman imputation and individual level multiple imputation may vary depending of the quantity and position of missing data. We chose to include these individual centred approaches as they were the only individualised approaches that were statistically equivalent to the true data across all activity types in simulation study 1. As in the first simulation study, we utilised 14-days (20160 minutes) of data for each participant. We simulated missingness randomly throughout the day and in all iterations, the maximum length of each insertion was set to 120 minutes. The simulations were split in to 10 windows of missingness, where the number of missing periods inserted for each participant increased incrementally with each simulation window. In the first window, the number of missing periods per participant was sampled from a uniform distribution between 0–10, the second between 10–20 up to the tenth which inserted 90–100 missing periods in each iteration. Within each window 20 simulations were conducted per participant, for a total of 21,800 iterations of each algorithm overall.

### Physical activity metrics

Each of the imputation methods tested in both simulation studies were used to address a number of distinct physical activity metrics including total steps, total daily energy expenditure (TDEE) and minutes of sedentary, light, moderate and vigorous physical activity. Both steps and TDEE for a given interval are extracted from the FC2 and time in each of sedentary, light, moderate and vigorous are defined by the heart rate reserve (HRR) method which is computed for each minute in the dataset. To facilitate this method, we estimated maximum heart rate for each participant using the Tanaka method; (208–0.7 x age) [39]. To define resting heart rate, we first determined sleeping heart rate, which was defined as the mean of the lowest 20 consecutive minutes observed between 00:00 and 08:00 am, when steps/min were < 5. After sleeping heart rate was defined, an 8% increase was used to approximate resting heart rate as this represents a typical difference between resting and sleeping heart rate [40]. Relative intensity of each minute was then calculated:

$$\%HRR=\frac{\left(HR-{HR}_{REST}\right)}{\left({HR}_{MAX}-{HR}_{REST}\right)}*100$$ (1)

The following cut points for were applied: Sedentary (<20% HRR), light (20–40% HRR), moderate (40–60% HRR), and vigorous (≥60% HRR) [32]. For each missing minute in the dataset, each of the imputation methods described above were used to impute or scale steps, caloric expenditure and heart rate to produce hourly, daily and average physical activity estimates.

### Statistical analysis

All data are presented as means and standard deviations unless otherwise stated and a flowchart detailing both simulation studies is available in S2 Fig. To evaluate the performance of each method, root mean squared error (RMSE) was calculated for all physical activity metrics for hourly, daily and 14-day averages, relative to the observed data. Where RMSE is defined as:

$$RMSE=\sqrt{\sum_{i=1}^{n}\frac{{\left({\hat{y}}_{i}-y_{i}\right)}^{2}}{n}}$$ (2)

Where ${\hat{y}}_{i}$ refers to predicted values, *y*_*i*_ refers to the true values and *n* refers to the number of observations. Equivalence tests were performed to investigate whether the models were statistically equivalent to the true data. To be considered equivalent, the 90% confidence interval of the estimate must fall within ± 10% of the criterion mean. Simulation study 1 was conducted on an intel i7-8750H with 32GB RAM and 12 logical processors. Simulation study 2 was undertaken on ARC3, part of the High-Performance Computing cluster at the University of Leeds, UK. Statistical analyses were conducted with R version 3.6.3 using a p-value of < 0.05 to determine statistical significance.

## Results

The participants meeting the minimum criteria were predominantly female (n = 93, male = 16) and were primarily from the Danish centre (DK = 69, UK = 23, Portugal = 17), Table 1 presents the demographic and physical activity results for the included sample.

**Table 1:** Demographic data and physical activity averages for the included sample (n = 109). Total daily energy expenditure (TDEE) is presented is kcals/day, sedentary, light, moderate and vigorous are presented in minutes/day.

	Mean ± SD	Minimum	Maximum
Age	47.46 ± 9.62	22	75
Height	1.69 ± 0.08	1.54	1.87
Weight	84.76 ± 15.59	50.5	148.4
BMI	29.64 ± 5	20.2	44.8
TDEE	2626.59 ± 504.66	1754.24	4492.25
Steps	10570.34 ± 3208.67	3202.50	19941.07
Sedentary	1087.76 ± 112.72	847.21	1284.64
Light	266.77 ± 94.83	102.29	484.14
Moderate	50.24 ± 31.6	6.43	132.86
Vigorous	7.29 ± 9.09	0.00	47.07

The computation time for each of the included algorithms in the first simulation were as follows: Overall mean imputation: 18.23 Minutes, Individual mean imputation: 1.27 Minutes, Overall multiple imputation: 17.61 Hours, Individual multiple imputation: 17.04 Minutes, Random forest imputation: 4.36 Hours, Kalman imputation: 2.16 Minutes, NoHoW method: 2.12 Seconds.

Table 2 illustrates the results of the first simulation study for 14-day, daily and hourly comparisons and Table 3 presents the results of equivalence tests for each of the methods. For TDEE, Individual multiple imputation had the smallest RMSE for 14-day (36.32 kcal), followed by the NoHoW method (39.51 kcal), and for the hourly comparison, Kalman imputation was superior (14.11 kcal). In the daily comparison the smallest RMSE was observed for the NoHoW method (115.86 kcal). All methods except removal (mean difference: -343.44 kcal) were statistically equivalent to the true data, with the smallest mean difference observed for Individual multiple imputation. For steps, the lowest RMSE was observed for the NoHoW method for 14-day (397.83 steps) and daily comparison (1366.92 steps) and Kalman imputation for hourly comparison (173.78 steps). All methods except removal (mean difference: -1320.74 steps, p-value >0.05), were statistically equivalent to the true data. In the HRR analysis, multiple imputation methods, Kalman imputation and the NoHoW algorithm were statistically equivalent for all sedentary, light, moderate and vigorous comparisons.

**Table 2:** Mean ± standard deviation estimates and Root Mean Squared Error (RMSE) for each of the imputation methods tested in simulation study 1. Total daily energy expenditure (TDEE) is presented is kcals, sedentary, light, moderate and vigorous are presented in minutes.

	Comparison		TRUE	Removal		Overall mean		Individual mean		Overall Multiple		Individual Multiple		Random Forest		Kalman		NoHoW	
TDEE	**14-day**		2626.59 ± 504.66	2283.15 ± 445.78		2645.66 ± 443.93		2645.49 ± 515.87		2649.59 ±457.11		2638.06 ± 513.24		2658.48 ±571.72		2660.96 ± 518.63		2653.61 ± 515.8	
		RMSE			351.27		71.30		40.97		67.52		36.32		117.37		49.57		39.51
	**Day**		2626.59 ±607.05	2283.15 ± 583.3		2645.66 ± 545.14		2645.49 ± 602.91		2649.59 ± 555.65		2638.06 ± 600.29		2658.48 ± 654.09		2660.96 ±632.27		2653.61 ± 627.21	
		RMSE			413.74		134.48		117.53		132.73		116.36		172.07		134.71		115.86
	**hour**		109.7 ± 65.24	100.12 ± 65.22		110.23 ± 61.53		110.25 ± 62.03		110.36 ± 61.7		110.01 ± 62.31		110.61 ± 63.2		110.69 ± 66.86		110.61 ± 67.58	
		RMSE			28.00		15.03		14.51		15.21		14.66		16.55		14.11		15.13
Steps	**14-day**		10570.34 ± 3208.67	9249.6 ± 2867.46		10718.22 ± 2860.93		10716.67 ± 3309.78		10741.09 ± 2817.02		10593.71 ± 3274.98		10049.5 ± 3472.95		10755.97 ± 3249.79		10791.34 ± 3309.09	
		RMSE			1412.90		519.53		426.36		591.06		398.18		864.48		432.66		397.83
	**Day**		10570.34 + 4775.05	9249.6 ± 4447		10718.22 ± 4360.78		10716.67 ± 4657.6		10741.09 ± 4343.52		10593.71 ± 4637.29		10049.5 ± 4804.18		10755.97 ± 5013.85		10791.34 ± 5009.86	
		RMSE			2049.36		1407.11		1368.44		1453.05		1387.16		1603.70		1588.95		1366.92
	**hour**		442.55 ± 738.52	405.61 ± 699.45		446.55 ± 694.48		446.58 ± 696.27		447.17 ± 695.47		442.93 ± 697.7		428.19 ± 700.15		448.23 ± 751.38		449.94 ± 763.31	
		RMSE			198.65		176.66		175.54		180.37		179.96		184.75		173.78		188.66
Sedentary	**14-day**		1087.76 ± 112.72	956.05 ± 101.01		1139.11 ± 109.98		1151.53 ± 105.13		1118.75 ± 100.63		1120.96 ± 110.35		1138.12 ± 112.52		1101.93 ± 117.73		1105.57 ± 116.03	
		RMSE			133.22		74.14		68.34		39.84		35.50		58.43		19.72		20.50
	**Day**		1087.76 ± 170.63	956.05 ± 174.15		1139.11 ± 160.95		1151.53 ± 156.73		1118.75 ± 152.3		1120.96 ± 157.97		1138.12 ± 165.97		1101.93 ± 175.74		1105.57 ± 173.68	
		RMSE			158.92		93.09		86.87		61.30		56.77		78.45		47.30		42.33
	**hour**		45.51 ± 17.82	41.93 ± 19.1		47.03 ± 16.83		47.38 ± 16.61		46.46 ± 16.44		46.54 ± 16.52		47.01 ± 17		45.99 ± 18.04		46.07 ± 17.89	
		RMSE			10.57		7.05		6.71		5.68		5.55		6.59		4.79		4.59
Light	**14-day**		266.77 ± 94.83	235.5 ± 84.53		247.25 ± 93.15		236.96 ± 86.51		264.11 ± 84.32		261.72 ± 91.8		247.05 ± 94.01		269.32 ± 96.42		274.66 ± 96.81	
		RMSE			34.02		48.07		35.76		20.66		11.20		30.39		10.63		10.98
	**Day**		266.77 ± 139.25	235.5 ± 127.82		247.25 ± 134.98		236.96 ± 129.6		264.11 ± 126.65		261.72 ± 131.03		247.05 ± 137.41		269.32 ± 143.71		274.66 ± 144.45	
					46.30		61.21		48.15		35.59		29.24		45.61		32.25		27.43
	**hour**		11.17 ± 14.15	10.33 ± 13.59		10.65 ± 13.86		10.36 ± 13.62		11.11 ± 13.47		11.03 ± 13.54		10.63 ± 13.92		11.2 ± 14.54		11.44 ± 14.69	
		RMSE			3.94		4.77		4.02		3.58		3.38		4.23		3.32		3.51
Moderate	**14-day**		50.24 ± 31.6	44.63 ± 28.42		44.63 ± 28.42		44.63 ± 28.42		48.81 ± 28.63		48.25 ± 31.18		44.65 ± 28.43		48.76 ± 31.17		52.22 ± 33.37	
		RMSE			6.83		6.83		6.83		4.90		3.90		6.80		3.81		3.79
	**Day**		50.24 ± 47.85	44.63 ± 43.95		44.63 ± 43.95		44.63 ± 43.95		48.81 ± 44.07		48.25 ± 45.54		44.65 ± 43.95		48.76 ± 49.15		52.22 ± 51.02	
		RMSE			11.38		11.38		11.38		10.20		9.68		11.38		12.73		10.29
	**hour**		2.11 ± 5.53	1.96 ± 5.29		1.96 ± 5.29		1.96 ± 5.29		2.07 ± 5.29		2.06 ± 5.3		1.96 ± 5.29		2.07 ± 5.67		2.18 ± 5.88	
		RMSE			1.31		1.31		1.31		1.29		1.28		1.31		1.49		1.59
Vigorous	**14-day**		7.29 ± 9.09	6.51 ± 8.38		6.51 ± 8.38		6.51 ± 8.38		6.92 ± 8.35		6.86 ± 9.13		6.51 ± 8.38		7.17 ± 9.03		7.55 ± 9.57	
		RMSE			1.44		1.44		1.44		1.29		1.28		1.44		1.00		1.16
	**Day**		7.29 ± 15.48	6.51 ± 14.61		6.51 ± 14.61		6.51 ± 14.61		6.92 ± 14.59		6.86 ± 14.99		6.51 ± 14.61		7.17 ± 15.66		7.55 ± 16.62	
		RMSE			4.05		4.05		4.05		4.03		4.05		4.05		4.03		4.62
	**hour**		0.3 ± 2.45	0.29 ± 2.36		0.29 ± 2.36		0.29 ± 2.36		0.3 ± 2.36		0.3 ± 2.37		0.29 ± 2.36		0.31 ± 2.52		0.32 ± 2.6	
		RMSE			0.47		0.47		0.47		0.48		0.48		0.47		0.52		0.60

**Table 3:** Mean ± standard deviation estimates and equivalence test results for each of the imputation methods tested in simulation study 1. Total daily energy expenditure (TDEE) is presented is kcals, sedentary, light, moderate and vigorous are presented in minutes. Bounds refers to the equivalence boundaries and p-value upper and lower refers to equivalence tests at the upper and lower equivalence bounds.

		TRUE	Imputed	Mean difference	Bounds	P-value lower	P-value upper
**TDEE**	Removal	2626.59 ± 504.66	2283.15 ± 445.78	-343.44	± 262.66	1	0
	Overall mean	2626.59 ± 504.66	2645.66 ± 443.93	19.08	± 262.66	0	0
	Individual mean	2626.59 ± 504.66	2645.49 ± 515.87	18.9	± 262.66	0	0
	Overall Multiple	2626.59 ± 504.66	2649.59 ± 457.11	23.01	± 262.66	0	0
	Individual Multiple	2626.59 ± 504.66	2638.06 ± 513.24	11.48	± 262.66	0	0
	Random Forest	2626.59 ± 504.66	2658.48 ± 571.72	31.89	± 262.66	0	0
	Kalman	2626.59 ± 504.66	2660.96 ± 518.63	34.37	± 262.66	0	0
	NoHoW	2626.59 ± 504.66	2653.61 ± 515.8	27.02	± 262.66	0	0
**Steps**	Removal	10570.34 ± 3208.67	9249.6 ± 2867.46	-1320.74	± 1057.03	1	0
	Overall mean	10570.34 ± 3208.67	10718.22 ± 2860.93	147.88	± 1057.03	0	0
	Individual mean	10570.34 ± 3208.67	10716.67 ± 3309.78	146.33	± 1057.03	0	0
	Overall Multiple	10570.34 ± 3208.67	10741.09 ± 2817.02	170.75	± 1057.03	0	0
	Individual Multiple	10570.34 ± 3208.67	10593.71 ± 3274.98	23.37	± 1057.03	0	0
	Random Forest	10570.34 ± 3208.67	10049.5 ± 3472.95	-520.84	± 1057.03	0	0
	Kalman	10570.34 ± 3208.67	10755.97 ± 3249.79	185.63	± 1057.03	0	0
	NoHoW	10570.34 ± 3208.67	10791.34 ± 3309.09	221	± 1057.03	0	0
**Sedentary**	Removal	1087.76 ± 112.72	956.05 ± 101.01	-131.71	± 108.78	1	0
	Overall mean	1087.76 ± 112.72	1139.11 ± 109.98	51.36	± 108.78	0	0
	Individual mean	1087.76 ± 112.72	1151.53 ± 105.13	63.78	± 108.78	0	0
	Overall Multiple	1087.76 ± 112.72	1118.75 ± 100.63	30.99	± 108.78	0	0
	Individual Multiple	1087.76 ± 112.72	1120.96 ± 110.35	33.2	± 108.78	0	0
	Random Forest	1087.76 ± 112.72	1138.12 ± 112.52	50.36	± 108.78	0	0
	Kalman	1087.76 ± 112.72	1101.93 ± 117.73	14.17	± 108.78	0	0
	NoHoW	1087.76 ± 112.72	1105.57 ± 116.03	17.81	± 108.78	0	0
**Light**	Removal	266.77 ± 94.83	235.5 ± 84.53	-31.27	± 26.68	1	0
	Overall mean	266.77 ± 94.83	247.25 ± 93.15	-19.51	± 26.68	0.046	0
	Individual mean	266.77 ± 94.83	236.96 ± 86.51	-29.8	± 26.68	0.949	0
	Overall Multiple	266.77 ± 94.83	264.11 ± 84.32	-2.65	± 26.68	0	0
	Individual Multiple	266.77 ± 94.83	261.72 ± 91.8	-5.05	± 26.68	0	0
	Random Forest	266.77 ± 94.83	247.05 ± 94.01	-19.72	± 26.68	0.001	0
	Kalman	266.77 ± 94.83	269.32 ± 96.42	2.55	± 26.68	0	0
	NoHoW	266.77 ± 94.83	274.66 ± 96.81	7.89	± 26.68	0	0
**Moderate**	Removal	50.24 ± 31.6	44.63 ± 28.42	-5.61	± 5.02	0.938	0
	Overall mean	50.24 ± 31.6	44.63 ± 28.42	-5.61	± 5.02	0.938	0
	Individual mean	50.24 ± 31.6	44.63 ± 28.42	-5.61	± 5.02	0.938	0
	Overall Multiple	50.24 ± 31.6	48.81 ± 28.63	-1.44	± 5.02	0	0
	Individual Multiple	50.24 ± 31.6	48.25 ± 31.18	-1.99	± 5.02	0	0
	Random Forest	50.24 ± 31.6	44.65 ± 28.43	-5.59	± 5.02	0.933	0
	Kalman	50.24 ± 31.6	48.76 ± 31.17	-1.48	± 5.02	0	0
	NoHoW	50.24 ± 31.6	52.22 ± 33.37	1.98	± 5.02	0	0
**Vigorous**	Removal	7.29 ± 9.09	6.51 ± 8.38	-0.78	± 0.73	0.672	0
	Overall mean	7.29 ± 9.09	6.51 ± 8.38	-0.78	± 0.73	0.672	0
	Individual mean	7.29 ± 9.09	6.51 ± 8.38	-0.78	± 0.73	0.672	0
	Overall Multiple	7.29 ± 9.09	6.92 ± 8.35	-0.37	± 0.73	0.002	0
	Individual Multiple	7.29 ± 9.09	6.86 ± 9.13	-0.43	± 0.73	0.005	0
	Random Forest	7.29 ± 9.09	6.51 ± 8.38	-0.78	± 0.73	0.672	0
	Kalman	7.29 ± 9.09	7.17 ± 9.03	-0.12	± 0.73	0	0
	NoHoW	7.29 ± 9.09	7.55 ± 9.57	0.26	± 0.73	0	0

**Fig 3:**
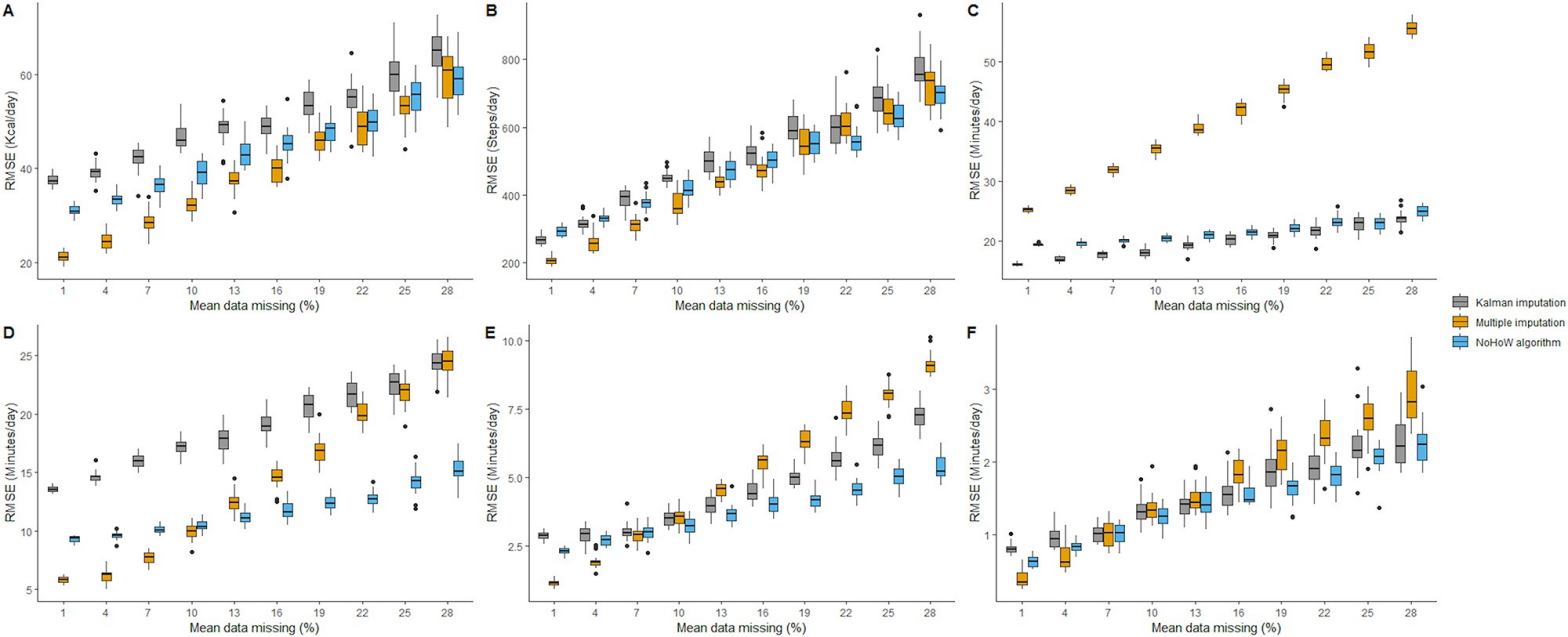
Boxplots detailing Root Mean Squared Error (RMSE) values from simulation study 2 for each window of missingness. Data are presented for TDEE (A), Steps (B), Sedentary (C), Light (D), Moderate (E), Vigorous (F). Mean missing data refers to the additional data added in the simulations.

In the second simulation study, which is visually represented as boxplots in Fig 3, the aggregated RMSE for each of the tested approaches tended to increase with the proportion of missing data. For the TDEE estimation (Fig 3A), the first iteration (1% missingness added) resulted in a mean RMSE of 31.14 kcal/day for the NoHoW method (range 28.82–33.12 kcal/day) compared to multiple imputation: 21.30 kcal/day (range 19.20–23.11 kcal/day) and Kalman imputation: 37.44 kcal/day (range 35.49–39.90 kcal/day). Comparatively, at the 10th insertion of missingness (~28% missingness added) a maximum RMSE of 68.89 kcal/day, 68.05 kcal/day and 72.55 kcal/day was observed for NoHoW, multiple imputation and Kalman imputation, respectively. For steps (Fig 3B), evidence of slightly superior performance was observed for multiple imputation at the lower levels of missingness (<19%). However, mean RMSE values for each of the methods remained similar and did not differ by more than 86 steps/day. In the HRR analysis, differences were the greatest in the sedentary comparison (Fig 3C), with the NoHoW and Kalman methods having a lower mean RMSE than multiple imputation at each window. The largest difference was observed at 28% missingness, where the mean RMSE values were 24.87 mins/day (range: 23.15–26.39 mins/day) for the NoHoW method, 55.56 (range 53.69–57.76) mins/day for multiple imputation and 23.73 mins/day (range 21.46–26.89 mins/day) for Kalman imputation. For light (Fig 3D) and moderate (Fig 3E) the NoHoW method showed the lowest mean RMSE values after 13% missingness. Its largest mean RMSE of 15.19 mins/day (range 12.81–17.42 mins/day) for light activity and 5.38 mins/day (range 4.72–6.26 mins/day) for moderate activity were observed at 28% missingness. Lastly, in the vigorous activity simulation (Fig 3F), multiple imputation had the lowest mean RMSE with <7% added missingness but Kalman and NoHoW methods were superior at higher levels of missingness. In the 28% missingness window, NoHoW reached a mean RMSE of 2.25 mins/day (range 1.84–3.03 mins/day) mins/day and Kalman reached 2.28 mins/day (range 1.85–2.95 mins/day). Results of the second simulation study are available in S1 Table.

## Discussion

The use of commercial activity monitors in research environments is proliferating, creating new research opportunities, however, it is critical to take steps to ensure the integrity of these data is not challenged by missing data. The purpose of the present study was to develop and test a methodology to account for missingness in physical activity data collected with a commercial activity monitor in a free-living environment. In our initial experiments, we utilised ICC analyses to show that if data are scaled within an hour, the relative data requirements to meet an ICC threshold of 0.9 are minimal (~5 minutes). This relates to the relative similarity between ‘local’ data points, as confirmed by our autocorrelation analyses. We also show that if the data are not scaled by wear time the relative requirements for a day equates to approximately 18 hours per day. This is in contrast to a previous study, which showed that relative to a 14 hours/day criterion, at least 13 hours/day of accelerometer data are required [41]. This slight discrepancy in the proportion of the day required may relate to the inclusion of night hours in our sample. Given the likelihood that this is a highly sedentary period, missing data at night is likely to be less influential on daily totals.

In simulation study 1, we used each of the tested methods to impute metrics that are likely to be of importance depending on the specific research aims. Our results suggest differential outcomes depending on the metric selected, for instance, random forest imputation, overall mean and individual mean methods did not impute vigorous or moderate minutes regularly, as reflected in the non-significant equivalent results (indicating these methods are not statistically equivalent). This is likely due to the low proportion of the day in which these activities are performed. In the first simulation study, we observed a slight tendency for the NoHoW method to overestimate minutes of moderate and vigorous activity. This may relate to the position of the missing data in simulation 1; For example, if missing data occurs in the sedentary period after an exercise bout then this period will be overestimated. As exercise is infrequent in non-athlete populations this is unlikely to result in a large error in mean differences. Indeed, the estimates for moderate and vigorous differed by < 2 minutes/day in the 14-day comparison. Researchers should consider imputation strategies based on observed activity data from their sample or should select methodologies which are statistically equivalent in the specific activities of interest.

We have also shown that all tested methods for all comparisons resulted in a RMSE which was lower than no imputation (i.e. removal). Making no attempt to adjust for missingness effectively assumes that activity was 0 and our results demonstrate the potential implications of this. In our first study, ~14% of the day was missing on average with ~12% inserted, equating to a wear time of 20–22 hours, which falls within the acceptable levels of missingness for most accelerometer research [14,15] and therefore evidences the importance of using one of these methods even in the case of relatively small quantities of missing data. Of the imputation methods tested, an advantage of individual-centred methods was observed, specifically Kalman imputation, individual multiple imputation and the NoHoW algorithm. Indeed, in our second simulation study, in which the maximal missingness approached double the quantity of our first simulation study the RMSE for TDEE was lower than the values observed for removal, overall mean and random forest imputation in simulation study 1, indicating the efficacy of these methods.

We simulated missingness evenly throughout the entire 24-hour period in relation to the observed patterns of missingness in the NoHoW trial. This is contrary to a previous study observing that missing data patterns more frequently occur at the beginning and end of the day [42]. It is of note that we utilised wrist-worn devices compared to the aforementioned study, which utilised hip worn accelerometers. Unlike wrist-worn monitors, hip-worn accelerometers are generally removed with changing of clothes. This may encourage compliance [43] and contribute to a more uniform distribution of missingness throughout the day.

We consider the relative computational simplicity of the NoHoW method to be a significant advantage. Accelerometer data of this kind can be extremely high volume and researchers must select their imputation strategy with consideration of both error reduction and computational feasibility. It may be possible to utilise advanced machine learning techniques to impute missing data, but these methods are computationally expensive and may be technically inaccessible to many researchers. In addition, more information (e.g. physiological, psychological or behavioural factors) may allow for more accurate multivariate imputation techniques but in free-living widescale settings this information is likely to be limited, thus our method is likely to be widely applicable. A further advantage of the present study is the testing of numerous activity metrics in addition to steps. Steps are a highly interpretable and relatable metric produced by wearable devices and some evidence suggests that estimates of steps from Fitbit devices are more valid and reliable than other derived variables, i.e. TDEE [44–46] although machine learning techniques may facilitate the refinement of energy expenditure estimates [47]. Nevertheless, the metric of interest to researchers will vary depending on the aims and hypotheses of a study and we demonstrate that the NoHoW method, Kalman imputation and individual level multiple imputation perform particularly well across a variety of physical activity metrics.

Key limitations of the present study are the utilisation of participants with a high proportion of wear time (>97.5%). Whilst highly adherent participants were required in order to have a near-complete dataset to validate against, we cannot rule out the possibility that the included participants are in some way behaviourally different from the participants that remove the FC2 more frequently. Second, we inserted missing data at random positions, and it remains uncertain how representative this is of free-living data in other studies. Participants may remove devices for comfort, aesthetic reasons, charging or under conditions where they would not wish to have measurements made (e.g. extreme sedentariness) and thus, it is possible that missingness is not completely at random [48] and may differ between populations and research studies. Unfortunately, no definitive method exists to test if data are missing at random [49] and many imputation strategies have limited capabilities to overcome this. However, our second simulation study simulates a wide variety of missing patterns in an attempt to identify such biases and worst-case scenarios in the selected methods.

Incorporation of activity monitoring devices is a necessary step in improving physical activity and energy balance tracking in research and clinical settings. We have proposed a simple and accessible methodology which effectively reduces the bias introduced to physical activity estimates by non-wear time and may improve the validity of research conclusions. Other imputation strategies (i.e. multiple imputation and Kalman imputation) performed comparatively well and importantly, all the methods tested in this study are superior to data removal. Researchers and clinicians utilising commercial activity monitors to monitor physical activity longitudinally should account for missingness in datasets and the algorithm presented in this study offers an approach to this.

## Supporting information

S1 Table Aggregated results for each window of missingness for each physical activity metric in simulation study 2. Difference refers to the difference in means between the imputed and true value. Abbreviations: SD: standard deviations, RMSE: Root mean squared error.
(DOCX)

S1 Fig The percentage of missing data for each hour of the day in the NoHoW trial. (DOCX)

S2 Fig A density plot detailing the lengths of missing data (<1440 minutes in length) in the NoHoW trial. The mean is represented by the red dashed line and the median is represented by the blue dashed line.
(DOCX)

S1 Data A flowchart detailing the simulation procedures conducted in this study. (PDF)
