# Correction: A novel scaling methodology to reduce the biases associated with missing data from commercial activity monitors

**DOI:** 10.1371/journal.pone.0238965

**Published:** 2020-09-03

**Authors:** R. O’Driscoll, J. Turicchi, C. Duarte, J. Michalowska, S. C. Larsen, A. L. Palmeira, B. L. Heitmann, G. W. Horgan, R. J. Stubbs

The following information is missing from the Funding statement: This project has received funding from an EPSRC Impact Acceleration Grant (Grant number: EP/R511717/1).

## References

[1] O’Driscoll R, Turicchi J, Duarte C, Michalowska J, Larsen SC, Palmeira AL, et al (2020) A novel scaling methodology to reduce the biases associated with missing data from commercial activity monitors. PLoS ONE 15(6): e0235144 10.1371/journal.pone.0235144 32579613 PMC7313747

