# Supplementary material for: A novel scaling methodology to reduce the biases associated with missing data from commercial activity monitors
Source: PLoS One. 2020 Jun 24;15(6):e0235144. doi: 10.1371/journal.pone.0235144 (PMC7313747; doi:10.1371/journal.pone.0235144)
Supplement: S1 Table — Difference refers to the difference in means between the imputed and true value. Abbreviations: SD: standard deviations, RMSE: Root mean squared error. (DOCX) [file pone.0235144.s001.docx]

|  |  | NoHoW |  |  |  |  |  |  | Multiple Imputation |  |  |  |  |  |  | Kalman |  |  |  |  |  |  |
| --- | --- | --- | --- | --- | --- | --- | --- | --- | --- | --- | --- | --- | --- | --- | --- | --- | --- | --- | --- | --- | --- | --- |
| **Metric** | **Window** | **Mean** | **SD** | **Difference** | **RMSE mean** | **RMSE median** | **RMSE minimum** | **RMSE maximum** | **Mean** | **SD** | **Difference** | **RMSE mean** | **RMSE median** | **RMSE minimum** | **RMSE maximum** | **Mean** | **SD** | **Difference** | **RMSE mean** | **RMSE median** | **RMSE minimum** | **RMSE maximum** |
|  |  |  |  |  |  |  |  |  |  |  |  |  |  |  |  |  |  |  |  |  |  |  |
| **TDEE** | **1** | 2653.42 | 0.95 | 26.83 | 31.14 | 30.94 | 28.82 | 33.12 | 2642.21 | 1.01 | 15.63 | 21.30 | 21.08 | 19.20 | 23.11 | 2658.34 | 0.95 | 31.75 | 37.44 | 37.29 | 35.49 | 39.90 |
|  | **2** | 2653.27 | 1.74 | 26.69 | 33.37 | 33.54 | 30.89 | 36.53 | 2639.92 | 1.80 | 13.33 | 24.63 | 24.42 | 21.95 | 28.31 | 2658.06 | 1.53 | 31.47 | 39.30 | 39.43 | 35.24 | 43.27 |
|  | **3** | 2652.90 | 2.19 | 26.31 | 36.52 | 36.55 | 31.54 | 40.64 | 2637.42 | 2.63 | 10.84 | 28.65 | 28.47 | 23.87 | 34.02 | 2658.35 | 2.43 | 31.76 | 42.20 | 42.46 | 34.15 | 45.32 |
|  | **4** | 2653.45 | 2.50 | 26.86 | 39.01 | 39.11 | 33.44 | 43.29 | 2634.83 | 2.64 | 8.25 | 32.33 | 32.23 | 28.66 | 37.27 | 2658.98 | 2.10 | 32.39 | 46.42 | 46.05 | 43.11 | 53.72 |
|  | **5** | 2653.59 | 2.65 | 27.00 | 43.30 | 42.86 | 39.59 | 50.00 | 2633.03 | 3.02 | 6.44 | 37.52 | 37.34 | 30.65 | 41.80 | 2657.87 | 2.95 | 31.28 | 48.60 | 49.33 | 41.11 | 54.41 |
|  | **6** | 2653.22 | 3.94 | 26.64 | 45.30 | 45.26 | 37.85 | 54.86 | 2630.24 | 4.50 | 3.65 | 39.75 | 40.02 | 36.01 | 44.82 | 2657.30 | 3.59 | 30.71 | 48.57 | 48.94 | 42.93 | 53.31 |
|  | **7** | 2652.13 | 3.60 | 25.55 | 48.05 | 48.59 | 43.40 | 53.25 | 2628.40 | 3.37 | 1.81 | 46.10 | 45.96 | 41.59 | 51.91 | 2658.16 | 3.39 | 31.58 | 53.54 | 53.27 | 47.40 | 58.81 |
|  | **8** | 2650.41 | 2.95 | 23.82 | 49.91 | 49.80 | 42.38 | 55.95 | 2622.22 | 3.73 | -4.37 | 48.78 | 48.99 | 43.34 | 57.54 | 2657.17 | 4.91 | 30.59 | 54.70 | 55.14 | 44.74 | 64.58 |
|  | **9** | 2653.40 | 4.35 | 26.81 | 55.51 | 55.69 | 47.55 | 61.92 | 2622.73 | 4.41 | -3.86 | 52.79 | 53.30 | 44.13 | 57.52 | 2658.04 | 5.00 | 31.45 | 59.39 | 59.85 | 51.06 | 70.90 |
|  | **10** | 2651.04 | 4.04 | 24.46 | 59.07 | 59.05 | 51.20 | 68.89 | 2619.06 | 3.90 | -7.52 | 59.20 | 60.90 | 48.71 | 68.05 | 2658.21 | 4.34 | 31.62 | 64.60 | 65.15 | 54.88 | 72.55 |
|  |  |  |  |  |  |  |  |  |  |  |  |  |  |  |  |  |  |  |  |  |  |  |
| **Steps** | **1** | 10805.61 | 13.05 | 235.27 | 291.90 | 291.66 | 271.60 | 317.28 | 10694.98 | 13.10 | 124.64 | 206.34 | 205.75 | 187.75 | 232.66 | 10763.98 | 13.12 | 193.64 | 267.59 | 266.09 | 246.41 | 296.26 |
|  | **2** | 10802.20 | 24.73 | 231.86 | 330.03 | 329.13 | 302.91 | 360.77 | 10660.89 | 22.33 | 90.55 | 260.79 | 256.41 | 224.08 | 338.79 | 10761.57 | 20.11 | 191.23 | 316.96 | 313.37 | 281.82 | 366.37 |
|  | **3** | 10800.53 | 26.97 | 230.18 | 377.08 | 376.18 | 328.60 | 434.21 | 10626.34 | 28.23 | 56.00 | 311.35 | 312.92 | 262.80 | 376.98 | 10768.55 | 30.02 | 198.21 | 388.04 | 394.35 | 322.77 | 427.97 |
|  | **4** | 10808.87 | 32.32 | 238.53 | 417.16 | 411.94 | 361.67 | 472.68 | 10590.14 | 34.71 | 19.80 | 372.87 | 358.84 | 308.90 | 443.73 | 10777.31 | 28.40 | 206.97 | 451.00 | 448.51 | 418.41 | 495.35 |
|  | **5** | 10811.60 | 30.96 | 241.26 | 474.52 | 472.84 | 420.78 | 527.34 | 10568.00 | 33.75 | -2.34 | 437.90 | 436.41 | 396.31 | 484.87 | 10766.82 | 34.30 | 196.48 | 498.83 | 497.95 | 443.08 | 571.46 |
|  | **6** | 10799.77 | 45.28 | 229.43 | 500.57 | 501.42 | 433.74 | 550.42 | 10521.42 | 49.32 | -48.92 | 476.90 | 470.89 | 410.51 | 582.31 | 10768.88 | 45.63 | 198.54 | 523.26 | 522.33 | 475.83 | 604.44 |
|  | **7** | 10801.73 | 52.73 | 231.39 | 550.51 | 550.33 | 494.67 | 606.38 | 10509.06 | 49.57 | -61.28 | 551.31 | 543.59 | 457.38 | 637.15 | 10774.76 | 44.13 | 204.42 | 595.22 | 588.24 | 511.02 | 681.06 |
|  | **8** | 10774.72 | 48.87 | 204.38 | 560.91 | 554.54 | 508.35 | 663.75 | 10427.40 | 59.47 | -142.94 | 611.23 | 601.73 | 551.35 | 762.66 | 10765.56 | 66.49 | 195.22 | 602.33 | 599.08 | 520.67 | 749.52 |
|  | **9** | 10809.24 | 55.23 | 238.90 | 629.84 | 625.40 | 560.61 | 704.13 | 10428.41 | 49.33 | -141.93 | 649.65 | 638.63 | 587.05 | 727.25 | 10778.26 | 74.12 | 207.92 | 690.48 | 686.65 | 580.70 | 829.83 |
|  | **10** | 10787.69 | 67.94 | 217.35 | 692.79 | 700.76 | 590.01 | 797.25 | 10387.58 | 51.53 | -182.76 | 718.92 | 738.28 | 618.82 | 844.70 | 10776.60 | 80.49 | 206.26 | 771.70 | 755.71 | 672.65 | 930.92 |
|  |  |  |  |  |  |  |  |  |  |  |  |  |  |  |  |  |  |  |  |  |  |  |
| **Sedentary** | **1** | 1105.81 | 0.24 | 18.05 | 19.40 | 19.40 | 19.04 | 19.83 | 1112.04 | 0.35 | 24.29 | 25.24 | 25.19 | 24.51 | 25.90 | 1101.75 | 0.27 | 13.99 | 16.15 | 16.16 | 15.74 | 16.66 |
|  | **2** | 1105.84 | 0.45 | 18.08 | 19.66 | 19.68 | 18.74 | 20.50 | 1115.04 | 0.51 | 27.29 | 28.50 | 28.40 | 27.52 | 29.37 | 1101.93 | 0.41 | 14.18 | 16.92 | 16.89 | 16.09 | 17.56 |
|  | **3** | 1105.92 | 0.41 | 18.16 | 20.08 | 20.15 | 19.15 | 20.88 | 1118.16 | 0.72 | 30.40 | 31.87 | 31.86 | 30.54 | 33.01 | 1102.02 | 0.69 | 14.27 | 17.69 | 17.81 | 16.73 | 18.41 |
|  | **4** | 1105.97 | 0.66 | 18.21 | 20.43 | 20.43 | 19.55 | 21.28 | 1121.32 | 0.84 | 33.57 | 35.49 | 35.50 | 33.48 | 36.95 | 1101.84 | 0.72 | 14.08 | 18.10 | 18.05 | 16.94 | 19.54 |
|  | **5** | 1105.87 | 0.67 | 18.11 | 20.97 | 20.97 | 19.80 | 21.93 | 1124.17 | 0.85 | 36.41 | 38.86 | 38.55 | 37.60 | 41.09 | 1102.40 | 0.98 | 14.64 | 19.32 | 19.34 | 16.97 | 20.84 |
|  | **6** | 1105.96 | 0.85 | 18.20 | 21.44 | 21.40 | 20.13 | 22.67 | 1127.17 | 1.23 | 39.42 | 42.06 | 42.27 | 39.48 | 43.82 | 1102.69 | 1.09 | 14.93 | 20.21 | 20.34 | 18.87 | 21.55 |
|  | **7** | 1106.10 | 0.76 | 18.34 | 22.04 | 22.00 | 20.54 | 23.70 | 1129.74 | 0.96 | 41.98 | 45.19 | 45.38 | 42.45 | 47.14 | 1102.47 | 1.27 | 14.71 | 20.85 | 20.90 | 18.91 | 22.17 |
|  | **8** | 1106.79 | 1.02 | 19.03 | 23.19 | 23.09 | 21.38 | 25.75 | 1133.91 | 1.18 | 46.15 | 49.67 | 49.45 | 48.27 | 51.61 | 1102.66 | 1.13 | 14.90 | 21.69 | 21.80 | 18.68 | 23.86 |
|  | **9** | 1105.58 | 1.13 | 17.83 | 22.94 | 23.10 | 21.07 | 24.62 | 1135.52 | 1.43 | 47.77 | 51.68 | 51.62 | 48.97 | 53.99 | 1103.27 | 1.55 | 15.52 | 22.92 | 23.00 | 20.12 | 24.79 |
|  | **10** | 1106.56 | 1.03 | 18.80 | 24.87 | 24.89 | 23.15 | 26.39 | 1138.91 | 1.01 | 51.15 | 55.56 | 55.56 | 53.69 | 57.76 | 1102.98 | 1.39 | 15.22 | 23.73 | 23.84 | 21.46 | 26.89 |
|  |  |  |  |  |  |  |  |  |  |  |  |  |  |  |  |  |  |  |  |  |  |  |
| **Light** | **1** | 274.67 | 0.19 | 7.91 | 9.30 | 9.34 | 8.73 | 9.60 | 270.29 | 0.28 | 3.52 | 5.80 | 5.79 | 5.30 | 6.24 | 278.66 | 0.26 | 11.90 | 13.54 | 13.55 | 13.14 | 14.02 |
|  | **2** | 274.58 | 0.38 | 7.81 | 9.60 | 9.64 | 8.72 | 10.23 | 268.08 | 0.42 | 1.31 | 6.20 | 6.29 | 5.04 | 7.36 | 279.13 | 0.48 | 12.37 | 14.62 | 14.60 | 13.82 | 16.02 |
|  | **3** | 274.60 | 0.34 | 7.83 | 10.11 | 10.03 | 9.51 | 10.82 | 265.93 | 0.60 | -0.84 | 7.70 | 7.73 | 6.63 | 8.52 | 279.76 | 0.62 | 13.00 | 15.96 | 15.97 | 14.90 | 16.98 |
|  | **4** | 274.46 | 0.46 | 7.69 | 10.44 | 10.32 | 9.53 | 11.39 | 263.64 | 0.66 | -3.13 | 9.93 | 9.96 | 8.18 | 11.05 | 280.56 | 0.66 | 13.79 | 17.13 | 17.26 | 15.70 | 18.51 |
|  | **5** | 274.57 | 0.63 | 7.80 | 11.19 | 11.06 | 10.12 | 12.36 | 261.60 | 0.68 | -5.17 | 12.45 | 12.43 | 10.77 | 14.50 | 280.68 | 0.93 | 13.91 | 17.82 | 17.91 | 15.65 | 19.86 |
|  | **6** | 274.49 | 0.69 | 7.72 | 11.73 | 11.63 | 10.47 | 13.36 | 259.57 | 1.01 | -7.20 | 14.53 | 14.54 | 12.46 | 15.95 | 281.23 | 1.03 | 14.46 | 19.11 | 18.90 | 17.10 | 21.23 |
|  | **7** | 274.52 | 0.55 | 7.75 | 12.39 | 12.37 | 11.29 | 13.60 | 257.82 | 0.72 | -8.95 | 16.90 | 16.89 | 14.92 | 19.96 | 282.05 | 1.24 | 15.29 | 20.70 | 20.78 | 18.32 | 22.27 |
|  | **8** | 273.95 | 1.00 | 7.18 | 12.74 | 12.70 | 11.55 | 14.18 | 254.63 | 1.05 | -12.14 | 20.11 | 19.81 | 18.33 | 21.90 | 282.51 | 1.19 | 15.74 | 21.63 | 21.67 | 20.01 | 23.61 |
|  | **9** | 274.84 | 1.08 | 8.08 | 14.18 | 14.24 | 11.86 | 16.32 | 253.69 | 1.23 | -13.07 | 21.73 | 22.01 | 18.96 | 23.77 | 282.56 | 1.50 | 15.80 | 22.50 | 22.68 | 19.90 | 24.15 |
|  | **10** | 274.35 | 0.93 | 7.58 | 15.19 | 15.11 | 12.81 | 17.42 | 251.46 | 0.99 | -15.31 | 24.51 | 24.48 | 21.41 | 26.54 | 283.66 | 1.25 | 16.89 | 24.41 | 24.32 | 21.89 | 26.33 |
|  |  |  |  |  |  |  |  |  |  |  |  |  |  |  |  |  |  |  |  |  |  |  |
| **Moderate** | **1** | 51.96 | 0.09 | 1.72 | 2.33 | 2.34 | 2.04 | 2.53 | 50.41 | 0.10 | 0.16 | 1.16 | 1.16 | 0.95 | 1.42 | 51.98 | 0.10 | 1.74 | 2.89 | 2.88 | 2.59 | 3.15 |
|  | **2** | 52.01 | 0.17 | 1.77 | 2.73 | 2.72 | 2.43 | 3.04 | 49.75 | 0.16 | -0.49 | 1.94 | 1.90 | 1.50 | 2.54 | 51.41 | 0.17 | 1.17 | 2.90 | 2.96 | 2.19 | 3.39 |
|  | **3** | 51.95 | 0.22 | 1.71 | 3.01 | 3.01 | 2.26 | 3.56 | 48.94 | 0.24 | -1.30 | 2.87 | 2.91 | 2.31 | 3.51 | 50.83 | 0.22 | 0.59 | 3.05 | 3.00 | 2.53 | 4.06 |
|  | **4** | 51.99 | 0.29 | 1.75 | 3.24 | 3.24 | 2.59 | 3.78 | 48.27 | 0.25 | -1.97 | 3.56 | 3.59 | 2.95 | 4.23 | 50.32 | 0.29 | 0.08 | 3.50 | 3.53 | 3.08 | 4.06 |
|  | **5** | 51.96 | 0.27 | 1.72 | 3.70 | 3.68 | 3.18 | 4.69 | 47.60 | 0.28 | -2.64 | 4.54 | 4.60 | 4.10 | 4.93 | 49.73 | 0.36 | -0.51 | 3.99 | 3.96 | 3.31 | 4.57 |
|  | **6** | 51.97 | 0.31 | 1.73 | 4.05 | 4.03 | 3.49 | 4.94 | 46.80 | 0.40 | -3.44 | 5.51 | 5.63 | 4.60 | 6.21 | 49.06 | 0.36 | -1.18 | 4.50 | 4.40 | 3.94 | 5.30 |
|  | **7** | 51.90 | 0.40 | 1.66 | 4.19 | 4.20 | 3.72 | 4.92 | 46.14 | 0.35 | -4.11 | 6.34 | 6.29 | 5.47 | 6.92 | 48.57 | 0.44 | -1.67 | 5.01 | 5.01 | 4.60 | 5.68 |
|  | **8** | 51.71 | 0.40 | 1.47 | 4.56 | 4.52 | 3.97 | 5.48 | 45.31 | 0.40 | -4.94 | 7.47 | 7.35 | 6.53 | 8.36 | 47.96 | 0.43 | -2.28 | 5.74 | 5.62 | 4.89 | 7.20 |
|  | **9** | 51.98 | 0.37 | 1.74 | 5.04 | 5.04 | 4.28 | 5.68 | 44.76 | 0.38 | -5.48 | 8.03 | 8.05 | 7.21 | 8.75 | 47.44 | 0.54 | -2.80 | 6.14 | 6.16 | 5.33 | 7.07 |
|  | **10** | 51.63 | 0.46 | 1.39 | 5.38 | 5.23 | 4.72 | 6.26 | 43.85 | 0.33 | -6.39 | 9.14 | 9.06 | 8.68 | 10.10 | 46.80 | 0.50 | -3.44 | 7.25 | 7.28 | 6.40 | 8.17 |
|  |  |  |  |  |  |  |  |  |  |  |  |  |  |  |  |  |  |  |  |  |  |  |
| **Vigorous** | **1** | 7.56 | 0.04 | 0.27 | 0.63 | 0.63 | 0.53 | 0.78 | 7.26 | 0.03 | -0.03 | 0.39 | 0.35 | 0.26 | 0.65 | 7.61 | 0.03 | 0.32 | 0.81 | 0.80 | 0.71 | 1.01 |
|  | **2** | 7.57 | 0.07 | 0.28 | 0.83 | 0.83 | 0.69 | 0.99 | 7.12 | 0.07 | -0.17 | 0.70 | 0.62 | 0.48 | 1.13 | 7.52 | 0.08 | 0.23 | 0.97 | 0.94 | 0.78 | 1.31 |
|  | **3** | 7.54 | 0.08 | 0.25 | 1.01 | 1.03 | 0.74 | 1.20 | 6.97 | 0.09 | -0.32 | 1.01 | 1.02 | 0.74 | 1.33 | 7.39 | 0.09 | 0.09 | 1.01 | 1.01 | 0.85 | 1.23 |
|  | **4** | 7.58 | 0.10 | 0.29 | 1.25 | 1.25 | 0.94 | 1.48 | 6.77 | 0.07 | -0.52 | 1.36 | 1.33 | 1.12 | 1.93 | 7.28 | 0.08 | -0.01 | 1.32 | 1.31 | 1.02 | 1.76 |
|  | **5** | 7.60 | 0.11 | 0.31 | 1.44 | 1.40 | 1.08 | 1.80 | 6.64 | 0.09 | -0.65 | 1.50 | 1.44 | 1.27 | 1.95 | 7.19 | 0.12 | -0.10 | 1.40 | 1.41 | 1.09 | 1.74 |
|  | **6** | 7.58 | 0.14 | 0.29 | 1.58 | 1.48 | 1.40 | 1.94 | 6.46 | 0.12 | -0.83 | 1.85 | 1.82 | 1.44 | 2.18 | 7.03 | 0.13 | -0.26 | 1.58 | 1.54 | 1.26 | 2.13 |
|  | **7** | 7.48 | 0.16 | 0.19 | 1.64 | 1.66 | 1.24 | 1.99 | 6.31 | 0.17 | -0.98 | 2.13 | 2.15 | 1.68 | 2.62 | 6.91 | 0.16 | -0.38 | 1.88 | 1.86 | 1.36 | 2.73 |
|  | **8** | 7.55 | 0.13 | 0.26 | 1.81 | 1.82 | 1.44 | 2.13 | 6.16 | 0.14 | -1.13 | 2.33 | 2.32 | 1.64 | 2.85 | 6.87 | 0.15 | -0.42 | 1.92 | 1.90 | 1.41 | 2.38 |
|  | **9** | 7.60 | 0.24 | 0.31 | 2.05 | 2.06 | 1.37 | 2.29 | 6.02 | 0.19 | -1.27 | 2.59 | 2.59 | 1.90 | 3.04 | 6.73 | 0.16 | -0.56 | 2.21 | 2.15 | 1.57 | 3.29 |
|  | **10** | 7.47 | 0.31 | 0.18 | 2.25 | 2.24 | 1.84 | 3.03 | 5.79 | 0.23 | -1.50 | 2.91 | 2.82 | 2.38 | 3.71 | 6.57 | 0.15 | -0.72 | 2.28 | 2.21 | 1.85 | 2.95 |

**S1 Table 1.** *Aggregated results for each window of missingness for each physical activity metric.* Difference refers to the difference in means between the imputed and true value

Abbreviations: SD: standard deviations, RMSE: Root mean squared error
