# Supplementary figures and images for: A novel scaling methodology to reduce the biases associated with missing data from commercial activity monitors
Source: PLoS One. 2020 Jun 24;15(6):e0235144. doi: 10.1371/journal.pone.0235144 (PMC7313747; doi:10.1371/journal.pone.0235144)

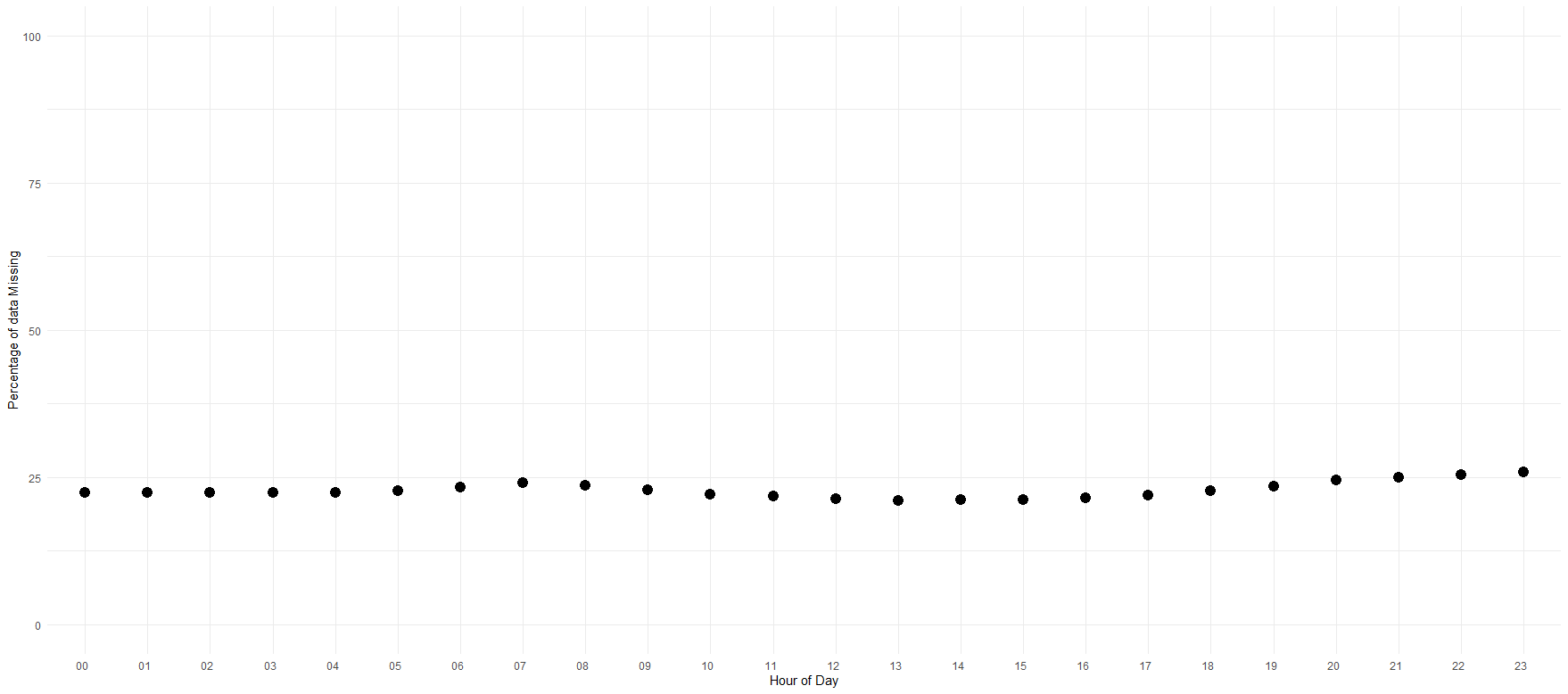


**S1 Fig 1.** *The percentage of missing data for each hour of the day in the NoHoW trial.*

Supplement: S1 Fig — (DOCX) [file pone.0235144.s002.docx]

## Simulation study 1

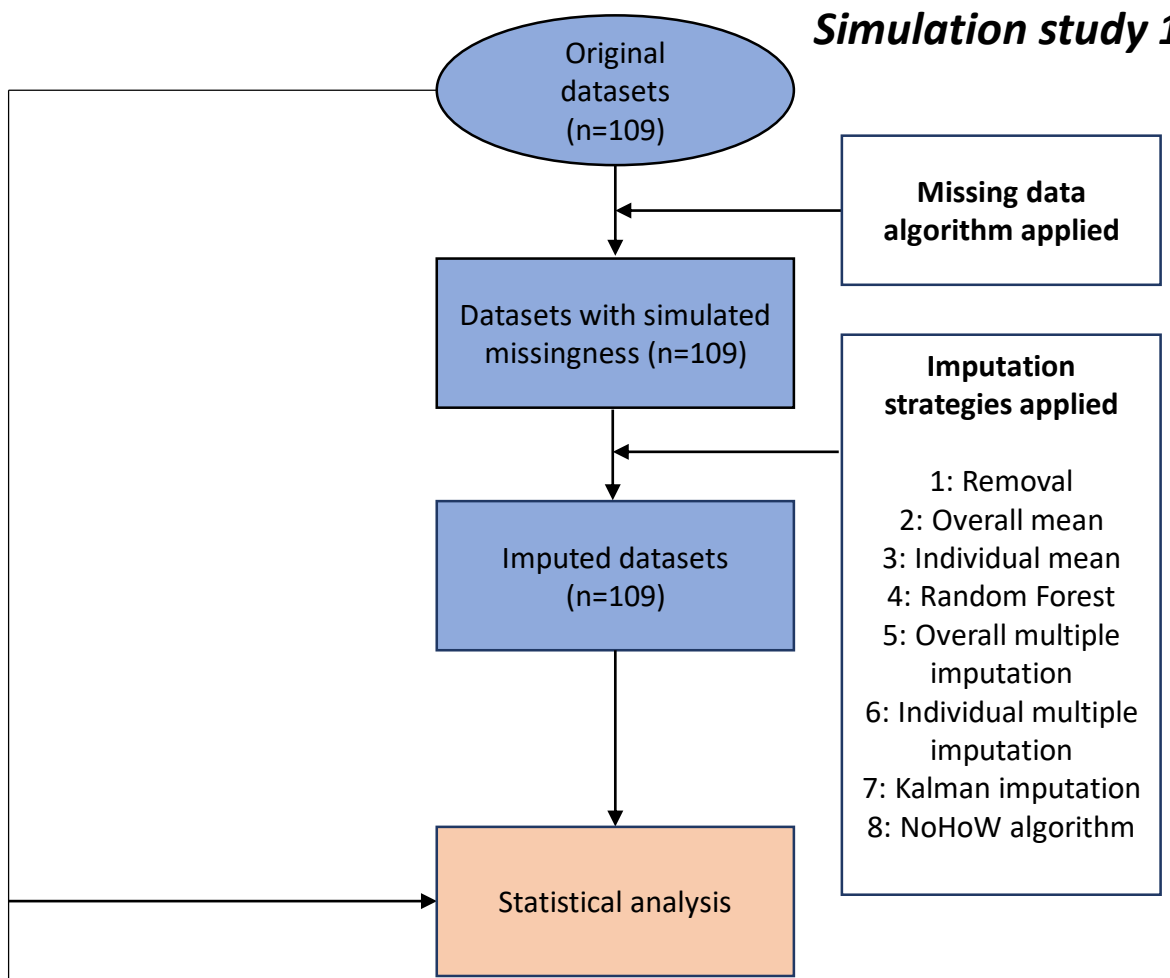

## Simulation study 2

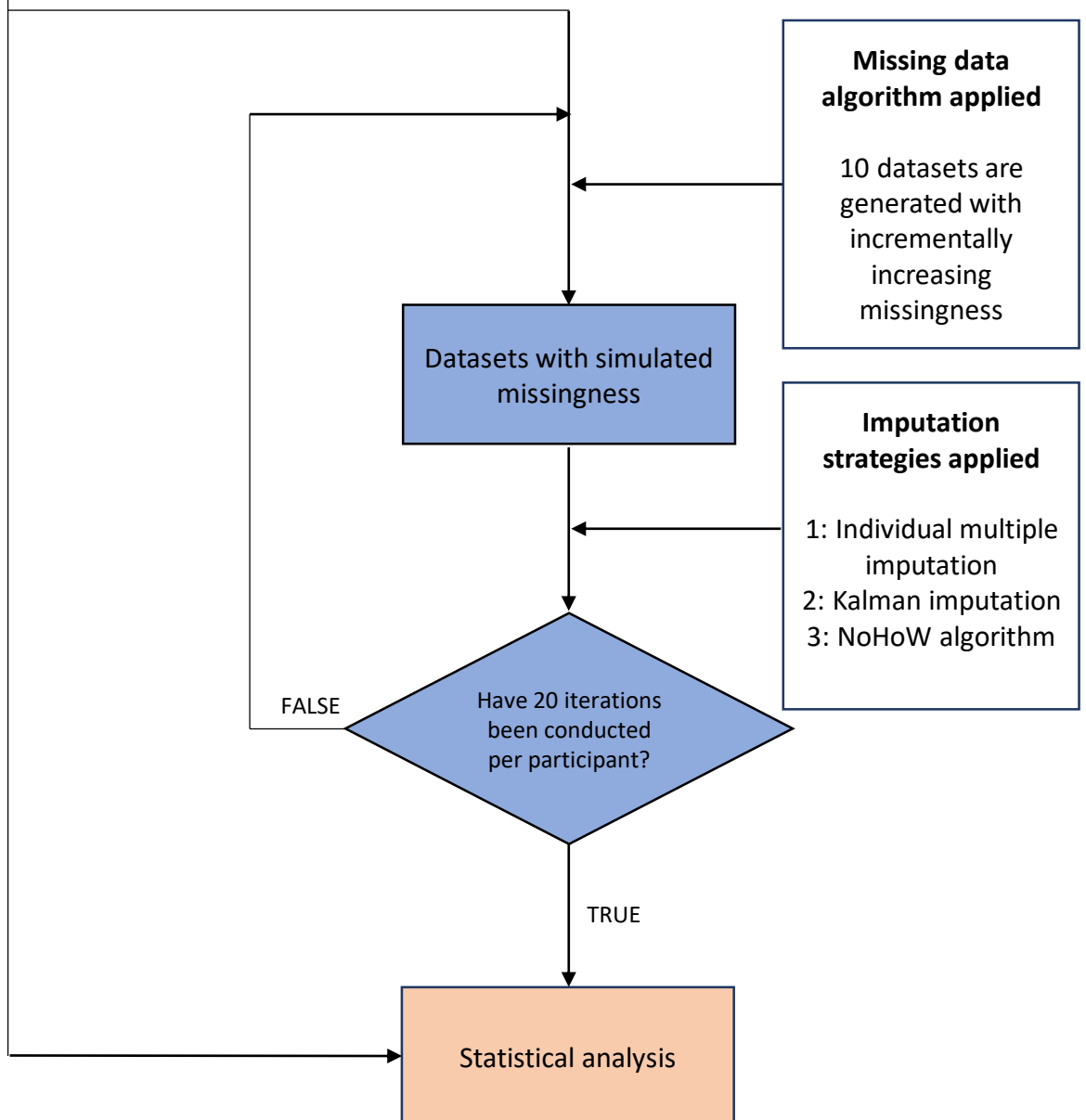

Supplement: S1 Data — (PDF) [file pone.0235144.s004.pdf]
