# Supplementary material for: A novel scaling methodology to reduce the biases associated with missing data from commercial activity monitors
Source: PLoS One. 2020 Jun 24;15(6):e0235144. doi: 10.1371/journal.pone.0235144 (PMC7313747; doi:10.1371/journal.pone.0235144)
Supplement: S2 Fig — The mean is represented by the red dashed line and the median is represented by the blue dashed line. (DOCX) [file pone.0235144.s003.docx]

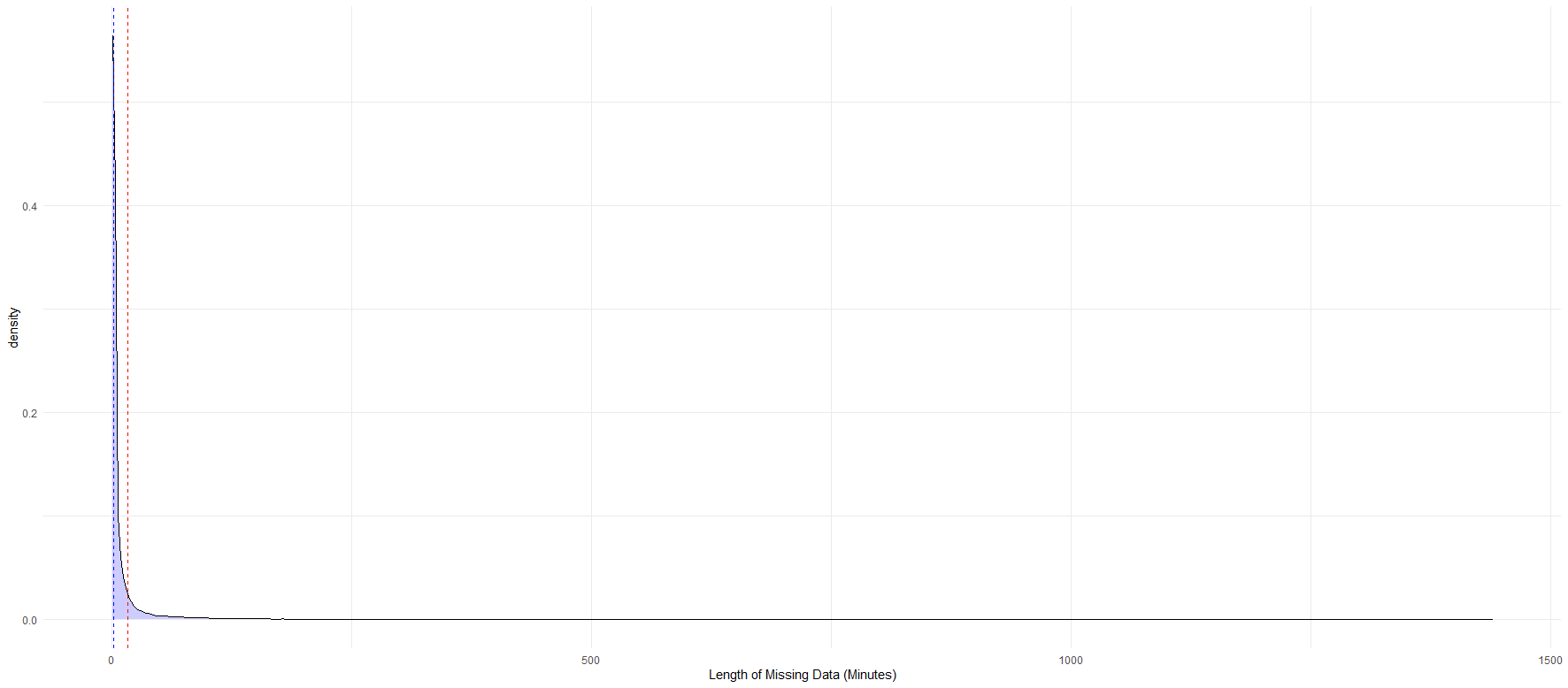


**S1 Fig 2.** *A density plot detailing the lengths of missing data (<1440 minutes in length) in the NoHoW trial. The mean is represented by the red dashed line and the median is represented by the blue dashed line.*
